# The Torsional Response of Civil Engineering Structures during Earthquake from an Observational Point of View

**DOI:** 10.3390/s21020342

**Published:** 2021-01-06

**Authors:** Philippe Guéguen, Ariana Astorga

**Affiliations:** ISTerre, Université Grenoble Alpes, USMB, CNRS, IRD, Université Gustave Eiffel, 38058 Grenoble, France; arilagua@gmail.com

**Keywords:** rotation, civil engineering, buildings, earthquake, Japan

## Abstract

This paper discusses the origins of torsion and its effect on the response of structures with a focus on the contribution of experimental data. The fact that torsion increases the stresses in structures, augmenting strain and damage during earthquakes, was confirmed in the 1960s. Over the years, the torsional response of structures has mainly been analysed through numerical studies, because few buildings are equipped with translational sensors, and even fewer are equipped with rotational sensors. This is likely to change as building instrumentation becomes more widespread and new generations of rotational sensors are developed. Therefore, this paper focusses on a number of scientific questions concerning the rotational response of structures during earthquakes and the contribution of experimental data to the understanding of this phenomenon.

## 1. Introduction

Since the 1960s and the emergence of earthquake engineering as a distinct field of engineering sciences, earthquake design for civil engineering structures has been concerned with the dynamic response induced by earthquakes. Analysis of a structure’s dynamic response is primarily motivated by the desire of earthquake engineers to reduce the consequences and losses of earthquakes; this is achieved by a conservative compromise involving the engineering simplification of current state-of-the-art scientific knowledge. For engineering seismologists, the analysis of strong ground motion is also motivated by the need to produce response spectra for design purposes via numerical or analytical methods, integrating the complexity of earthquake ground motion. Designing a building under seismic loading becomes extremely complicated, as this objective encompasses all the complexities inherent to seismic loading and the transfer of design loads to the structure, considering different scientific aspects that are sometimes closely related.

Among these aspects, rotational response is a key issue to be considered, as rotation can make a significant contribution to the response of most buildings during earthquakes. Today’s building codes prescribe the ultimate rotational capacity of structural elements (for example, among others, [1]), an aspect of rotation not addressed in this study, as well as the abundant literature on wind-induced rotational effects on structure response (among others [2,3]). We focus more on the seismic behaviour of a structure modelled by a lumped-mass system or a homogeneous continuous beam. In an equivalent 3D dynamic system, these rotations, of multiple origins, concern the following:

- rotations around the vertical axis (or torsion) due to the symmetric or asymmetric design of the building;
- rotations around the horizontal axis (or rocking) due to the inertial translational response of structures under earthquake loading, coupled with soil–structure interaction.

The rotational response of buildings under seismic loading makes their design much more complicated than the design of buildings considering a single translational response. The consideration of rotation in design codes is not recent. In particular, Rutenberg [4] and more recently Anagnostopoulos et al. [5] provide a complete review of the ins and outs of considering torsion in asymmetric structures from an engineering design point of view. Concerning rocking, the horizontal translational components of ground motion generate horizontal bending coupled with rotations around the horizontal axis, due to soil–structure interaction (SSI) phenomena, an exhaustive review of which can be found in Kausel [6]. Modern codes, such as Eurocode 8, Part 6 [7] and New Zealand’s seismic provision [8] emphasise the need to integrate SSI through specific studies, essentially by numerical modelling. Furthermore, for the design of special buildings, Eurocode 8, Part 6 [7] also recommends taking into account secondary effects due to the rocking seismic motion, using the response spectrum-based empirical method [9,10]. Rocking and torsion response spectra are both defined based on the properties of the uppermost 30 m layer of soil (i.e., Vs30) and the dynamic modal parameters of structures in translation (i.e., fundamental period and associated damping). These considerations remain arbitrary, and Anagnostopoulos et al. [5], similar to Rutenberg in 1992 [4] (as if no progress was made over two decades) for torsion and Zembaty [9] for rocking, found that the treatment of rotation by modern codes varies considerably because of the simplifications used. In the absence of observations that improve understanding, some reductive hypotheses have been proposed, which has led to oversimplified formulations of rather complex phenomena.

For years, rotational damage patterns observed on archaeological ruins or cultural heritage structures have been considered as evidence of seismic destruction due to high earthquake intensities [11]. Over the past three decades, scientific progress has mainly come from numerical and analytical models, although some pioneering instrumental observations have confirmed the role and origins of these rotations in seismic damage patterns. As stated by Trifunac et al. [12] and recently confirmed by Astorga et al. [13,14,15], significant progress in the understanding of the dynamics of buildings and the physics of the processes activated during earthquakes can only be achieved by generalising experimental recordings in more instrumented structures, in spite of the development of increasingly sophisticated numerical models. With regard to rotation, Lee and Trifunac [16] noted that despite recurrent engineering studies that have shown the importance of rotations in the response of buildings, the development and deployment of specific strong motion instruments to record rotations have remained somewhat rare.

Progress may now accelerate, mostly thanks to the development of new instruments from a wide variety of disciplines. For example, technologies are based on mechanical sensors, solid-state sensors, inclinometers, fiber-optic sensors, gyroscope-based sensors, and ring laser-based sensors. The most important features to make rotation sensors useful for seismology or earthquake engineering is their sensitivity to resolve weak-to-strong rotation motion (from around 0.01 rad/s at short distance to the seismic sources to 10^−6^ μrad/s for a tele-seismic event) and the ability to record the uncoupled rotation [17]. The seismological community has been aware of the importance of this field for some time now. Over the past three decades, seismologists have invested in the observation and interpretation of the rotation components of the seismic wave field, thanks to the development of dense networks (among others [18,19,20,21]), and the emergence of new rotation sensors (e.g., [22,23,24,25,26,27]). These sensors have gradually become more sensitive and broadband to record earthquake-induced rotational ground motion in the near field and far field. Since the seminal numerical study of Bouchon and Aki [28] on ground motion rotation, this renewed interest has been encouraged by experimental observation and associated signal processing and inversion schemes. That includes new rotational and strain observables to improve our understanding of earthquake processes and the structure of the Earth’s crust [29]. Two monographs [30,31] have been published, several working groups have been formed [32,33], and special issues on rotation have been published in seismological and earth science journals [29,34,35]. In spite of their evocative titles, these special issues contain very few papers related to the field of earthquake engineering, even though all the authors argue the need for earthquake-resistant structures. A few examples of buildings instrumented with sensors allowing rotation analysis do exist, but they are still marginal. Similar to seismology, progress in instrument deployment in structures could provide new observations supported by the development of innovative signal processing methods to improve the understanding of rotational structural response. This may result in new perspectives to refine the principles and foundations of the rotational dynamics of structures, addressing the fields of earthquake engineering and Seismic Structural Health Monitoring (S^2^HM, [36,37]).

The main objective of this paper is to discuss the contribution of earthquake data recorded in a structure in order to specify some properties of the rotation around a vertical axis. For this purpose, it is organised in two main parts: (1) a first one presenting the origin of rotation and the main results from theoretical or numerical studies that could be advantageously confirmed or improved by the contribution of experimental data; and (2) an example of torsion calculated in a Japanese building that not only is instrumented by an accelerometric array allowing the validation of several hypotheses but also has recorded a large number of earthquakes over 20 years. This paper begins with a brief review of the origins of rotations, particularly rotations arising from static eccentricity, and their consideration in seismic codes. Then, the notion of accidental eccentricity at the origin of the torsion that is often prevalent in the structural responses is described. Examples of experimental observations of torsion in structures and their numerical counterpart are provided in the fourth section, most of them using array-derived methods based on translation networks described in the same section. Finally, some aspects of accidental eccentricity are discussed using the example of a Japanese building that has been instrumented for more than 20 years and has recorded a large number of earthquakes. Finally, some perspectives for the experimental observation of rotation are discussed.

## 2. The Primary Origin of Torsional Motion in Buildings Linked to Static Eccentricity

**Figure 1:**
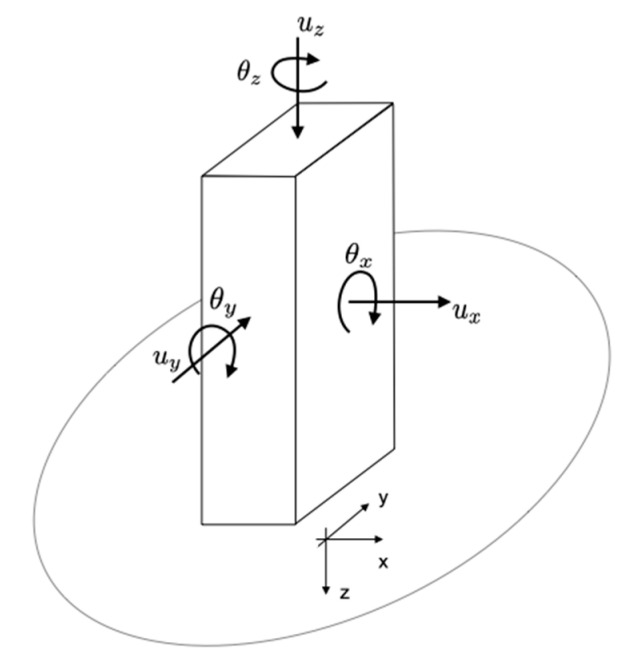
Six-component responses of civil engineering structures.

The literature uses many terms to describe rotations (e.g., twist, heading, torsion, eccentricity, and yaw for vertical axis rotations, and tilt, rolling, overturning, pitching, and rocking for horizontal axis rotations), and it proposes a partial definition of the mechanisms behind the rotations that can be expected in structures. This article applies current standard practice for structures related to the simplified model traditionally considered (Figure 1) with six degrees of freedom: (1) one vertical translation along the z-axis $u_{z}$ and two horizontal translations $u_{x}$ and $u_{y}$ along the x and y axes of the structure; (2) one rotation $\theta_{z}$ around the vertical axis (or torsion); (3) and two rotations $\theta_{x}$ and $\theta_{y}$ around the horizontal axes x and y (or rocking). Herein, only torsion rotations that are inherent to the building design or due to the seismic input ground motion are discussed.

Torsion is traditionally related to what the codes refer to as eccentricity in asymmetric structures. Table 1 is an attempt to qualitatively resume the origin and the effect of the torsion on the earthquake building response, according to some general structural features. Asymmetry, or symmetry, describes the distribution of masses and rigidities in the structure, and it does not refer to the geometry in plane or in elevation. Mallet [38] was probably one of the first to describe the residual rotational motions observed on monuments after historical earthquakes in terms of eccentricity of the centre of mass (CM) and the centre of rigidity (CR). This eccentricity means that the translational and rotational vibrations are coupled, i.e., the CM and CR of at least one storey do not coincide. The resulting inertia forces (acting at the CM) and elastic forces (acting at the CR) form a dynamic torque that interconnects the translational and torsional responses (such buildings are referred to as torsionally coupled) [39] activated by horizontal seismic ground motion only. Ayres [40,41] confirmed Mallet’s observations and the torsionally coupled building response with laboratory experiments on asymmetric, reduced scale buildings subjected to translational motions, with maximum rotation appearing at the resonance frequency of the translational modes.

Asymmetry makes structures more vulnerable to earthquakes. Before the advent of modern codes, provision codes required the effects of torsion due to so-called static eccentricity *e_s_* to be taken into account by applying additional equivalent lateral forces at a distance *e_s_* from the centre of stiffness [4,42]. The resulting torque along the height of the structure, in addition to shear and overturning forces, has a direct effect on the peak ductility demand of the resisting elements and the maximum lateral floor displacement [43]. In his review, Rutenberg [4] also noted many conflicting conclusions in almost all referenced studies due to model dependence. This is also confirmed by Paulay’s works [44,45] related to (among others) the elastic/ductile building behaviour considered in earthquake code design. Rutenberg [4] concluded on the absence of experimental confirmation of the rotation and a lack of instrumentation.

Over the years, seismic codes have been improved by transferring parts of new scientific knowledge on other origins of rotational structural motion, magnifying the static eccentricity between the CM and the CR of the structural system. As early as 1958, Housner and Outinen [46] reported that the dynamic effects of earthquakes (Table 1) significantly amplify static eccentricity by a coefficient α (e.g., α = 1.5 in EC8). Bustamante and Rosemblueth [47] introduced the notion of dynamic eccentricity, as opposed to static eccentricity, which amplifies the dynamic torques along the building height, which was later verified on more sophisticated building models [43,48,49,50,51,52]. This dynamic coefficient, applied in practice to simplify analytical analyses (e.g., static analyses), has generated many model-oriented scientific papers. In the absence of an intensive programme to record the rotational component in structures, the advent of modern dynamic numerical models has meant that this subject has ceased to attract attention, and modern codes only address the second term of Equation (1), i.e., the accidental eccentricity detailed below.

However, dynamic eccentricity (Table 1) may arise by coincidence or near coincidence of the natural frequencies of the torsion and translation modes [53]; this occurs in different ways under strong motion, since the translation and rotation modes are not proportional in the inelastic state [54]. Non-linearities in the earthquake responses of structural elements also modify the dynamic amplification of torsion under strong motion [55,56]. Moreover, in an asymmetric building, with coupled torsion, the position of the CR must be determined. However, Ivanovic et al. [57] observed a storey-dependent variation of CR between ambient vibrations and earthquake loadings. Finally, in a symmetric building, uncoupled torsion is to be expected; i.e., the CMs and CRs of all floors are on the same vertical axis and the translation and torsional responses are therefore independent [39]. Non-linear effects can be activated, leading to a significant reduction in the resonant frequency of structures (e.g., [13,14,15,58]), modifying dynamic uncoupled or coupled torsion in symmetric or asymmetric buildings [39]. All these results raise the question of how the rotational dynamic component, through the non-linear effects and the coupling of translation and torsion modes, is activated during earthquakes in actual symmetric and asymmetric buildings.

## 3. Accidental Eccentricity

Other additional forces are likely to occur due to a so-called accidental eccentricity *e_a_*, which may be multiple and difficult to separate with any degree of precision. Modern codes (e.g., [7]) introduce design eccentricity *e_d_*, considering other terms in addition to static eccentricity, as follows:

(1) $$e_{d}=\alpha \;e_{s}\;+\;\;e_{a}$$

where α represents the dynamic effects and *e_a_* is a general term to include any other source of eccentricity. The adjective “accidental” suggests that this eccentricity is the result of discrepancies between the design and the construction of the structure, implying that a building whose plane is nominally symmetric is in fact asymmetric to an unknown degree and undergoes torsional vibrations when subjected to pure translational ground motion. Accidental eccentricity is given as follows:

(2) $$e_{a}=\;\beta \;L.\;$$

Modern codes assign values between 0.05 and 0.1 to the β coefficient applied to the dimension perpendicular to the direction of the seismic motion L, which is a coefficient that varies according to design. The β value was based on expert judgement, and it is now accepted that accidental eccentricity is greater in symmetric than in asymmetric buildings, since the inherent eccentricity of the design (position of CM and CS) is prevalent in the rotational response of the latter. However, the origin of this accidental eccentricity has since been discussed in many publications.

### 3.1. Discrepancies between the Calculated and the Actual Values of the Stiffness and Mass of Structural Elements

For example, Chandler and Duan [59] argued that it is incorrect to consider accidental eccentricity in codes by ignoring design uncertainties, while De La Llera and Chopra [60] concluded that the increase in structural deformation due to uncertainty in stiffness is much smaller than the accidental torsion provisions (β value). Actually, De La Llera and Chopra [61] reported that the uncertainty of the design related to stiffness and mass distribution leads to an increase of structural member deformations in the order of 10% and 5% for RC and steel buildings, respectively (Table 1).

### 3.2. The Synchronised Orthogonal Components of Horizontal Ground Motion

In symmetric and asymmetric buildings (Table 1), the ground motion in both horizontal directions modifies the dynamic response of the structure through the coupling of the translational and rotational modes of vibration [62]. In particular, Chopra and Goel [63] and Lin and Tsai [54] showed that due to the complex inelastic coupling between the rotational and the two horizontal components, it is difficult to analyse the response of asymmetric structures under a bi-directional base motion using numerical methods. They proposed simplified engineering solutions for specific building features (e.g., resonance frequency, site conditions) and earthquake characteristics (e.g., magnitude, distance, focal mechanisms). They considered only a small number of examples, without considering the simultaneous actions of horizontal components, and considering asymmetric structures about one axis, and not the non-linear shift of the co-seismic resonance frequency that varies from one earthquake to another.

### 3.3. Rotation of Ground Motion at the Base

We can distinguish two processes, among others, at the origin of torsional ground motion. They are generally difficult to separate because their effects on the structural response are superimposed and similar. The first is the result of incoherent ground motion resulting from source (Table 1), propagation, or particular site condition effects, whereas the second results from wave passage and occurs when different points on the foundations are excited by the same motion but out of phase [5].

There are very few experimental examples of the first process on actual structures, with the exception of very large structures, such as bridges, where the distance between piers favours differential motion [64,65]. Differential longitudinal displacements can be twice as large as incoming waves [62], which might explain the collapse of bridges during the San Fernando (1971) and Miyagi-Ken-Oki (1978) earthquakes. The impact of differential motion on a simple structural model has been the subject of many numerical and analytical analyses [66,67,68], especially in the near field of seismic sources. In such cases, the induced differential motion and rotation considerably modify the response spectra, tending towards the classic spectral shape of horizontal motion in the far field [68]. Differential motion occurs both during earthquakes and under ambient noise, and it is exacerbated when the structures lie in a sedimentary basin. In this case, significant lateral variations at short wavelengths and reflections at the edges of the basin can generate a complex wave field with superimposed direct body waves and diffracted Rayleigh and Love waves, which are composed of a multitude of different phase-shifted waves and azimuths (e.g., [69,70,71,72]). Moreover, in urban areas, structures excited by natural vibrations (seismic noise) or by anthropogenic (traffic) and external loads (wind) cause diffracted waves back into the ground similar to surface waves [73,74]. These are out of phase at a given point on the basin surface, depending on the distance between buildings. Compared with the incoming wave field, the radiated energy becomes non-negligible in an urban environment when considering building assets [73] or a cluster of buildings [75], and it may affect the actual response of the building.

The second process (Table 1) is the consequence of the rotational components of seismic ground motion due to the displacements induced by the passage of seismic waves; this mostly concerns the contribution of SH and Love waves with regard to torsion [76]. Since the first theoretical [77], numerical [28], and experimental [78] publications, it has been confirmed that the rupture of the source along faults (inherent complex) generates a rotation field around a vertical axis. An important step in considering torsion in structural response has been the development of (abundant) studies on soil–structure interactions. In particular, Reissner and Sagoci [79] considered the vertical and torsional motion of a rigid mass with a circular base resting on an infinite half-space, Bycroft [80] proposed a solution of a rigid body on an infinite half-space for torsional motion; Apsel and Luco [81] provided an exact solution for the torsional response of foundations embedded in an elastic half-space, subjected to harmonic torque around the vertical axis, and for SH waves propagating in arbitrary directions; and Luco [82] expressed the elastic response in torsion of a structure with rigid circular foundations resting on an elastic half-space and subjected to SH waves with oblique incidence. Housner [83] was perhaps the first to demonstrate that this phenomenon, which is now called kinematic interaction, causes decreased effective motion in the vicinity of a relatively rigid and tall structure, due to the fact that the rigid structure cannot adapt to the deformations of the soil caused by waves of a wavelength less than the dimensions of the foundation. This highlights the frequency dependence of this phenomenon.

This pioneering work inspired Newmark [84], who examined the torsional response of symmetric structures caused by waves passing under the foundations. He called this phenomenon the τ effect, considering it to be a consequence of the kinematic interaction and the time delay of the wave passage at the different points of the foundations, assuming a constant and horizontal plane wave propagation velocity for embedded foundations. He proposed an engineering relation between the torsion and translation components of ground motion for the computation of displacements of symmetric Single-Degree-of-Freedom (SDOF) structures. Chandler and Duan [59] and De La Llera and Chopra [60] concluded that to consider accidental eccentricity in codes for dynamic inelastic analysis without the torsional ground motion is wrong. Displacements and strains in structural members can increase, particularly in flexible structures. De La Llera and Chopra [60] also reported differences between the calculation of a structure torsion response according to Newmark’s simplified method [84] and that directly derived from accelerometric recordings. This was attributed to the fact that Newmark’s approach assumes that the maximum rotation and translation of a symmetric building occur at the same time, which is a hypothesis that similar to others can only be verified experimentally.

Newmark’s study paved the way for more sophisticated solutions (examples among others: [18,76,85,86,87]), from horizontal motion recorded in a free field to the generation of products (such as synthetic seismograms and response spectra) for designing structures. Numerical studies, whether based on the analysis of equivalent static lateral forces (force applied at a distance—eccentricity—from the centre of stiffness) or dynamic analysis, conclude that design forces increase due to accidental eccentricity [60]. Overall, the effect of ground torsional motion on structures has rarely been investigated experimentally. The effects decrease rapidly with distance from the source (e.g., [18,28,71]) as do the effects on response spectra (e.g., [18,68]). For this reason, few buildings are instrumented with translation sensors that respond to this remote source site configuration, and even fewer are equipped with pure rotation sensors.

## 4. Experimental and Numerical Observation of Torsion

### 4.1. Experimental Observation

The rotational response of structures has long been mentioned as a major cause of seismic damage. As early as 1846, Mallet [38] insisted on the need to understand the phenomena that cause damage, “enabling them to pass by that which is accidental, and to apply right methods and suitable instruments in ascertaining the really important elements of their motions in measures of time and space, by which hereafter the narratives of earthquakes, ceasing to be merely scrolls written with mourning and lamentation and woe, may become systematised records of facts, which a true theory shall make available, and in the highest degree valuable, for the advancement of terrestrial physics and geology,” and particularly the vertical axis rotation of structures observed during the earthquakes of the 18-19th centuries (Boston earthquake, 1755; Calabria 1783 and Valparaiso 1783). In 1884, during the Peldon earthquake (UK), Stanley [88] noted a curious effect of earthquakes, which he attributed to an apparent rotation of the shock evident in many buildings. Hogdson [89], Ulrich [90], Rouse and Priddy [91], and Thornton [92] reported rotations observed on structures (chimneys, gravestones, etc.) during North American earthquakes. As early as 1925, Dewell [93] referred to Omori’s work on the rotation of structures with considerations according to the dimensions and periods of vibration of the structures. Thornton [92] attributes to the distribution of loads the tendency of structures to rotate around the vertical axis. These examples are not unique, and most of them are referenced by Sargeant and Musson [94] and Hinzen [95], who concluded that this rotation, resulting from the inherent design of the structure or the rotational ground motion, accentuates seismic damage.

With the advent of instrumental seismology, there is no shortage of examples of the prevalence of the torsional element in the response of structures: Hart et al. [96] used ambient vibration and earthquake data from the San Fernando earthquake (1971); Safak [97] observed a torsional response representing 10% of the translation in a tall steel building caused by the Loma Prieta earthquake; Li and Mau [98] attributed the damage caused by the Loma Prieta (1989) and Whittier Narrow (1987) earthquakes to the torsional response triggered by rotational ground motion, even in apparently symmetric buildings; Todorovska and Trifunac [99] attributed the significant ground level torsional response and the coupling between horizontal and torsional responses to irregularities in ground floor stiffness; Lemnitzer et al. [100] observed significant rotations with respect to translational motion in concrete buildings during the aftershock sequence of the Maule earthquake (Chile, 2010).

**Figure 2:**
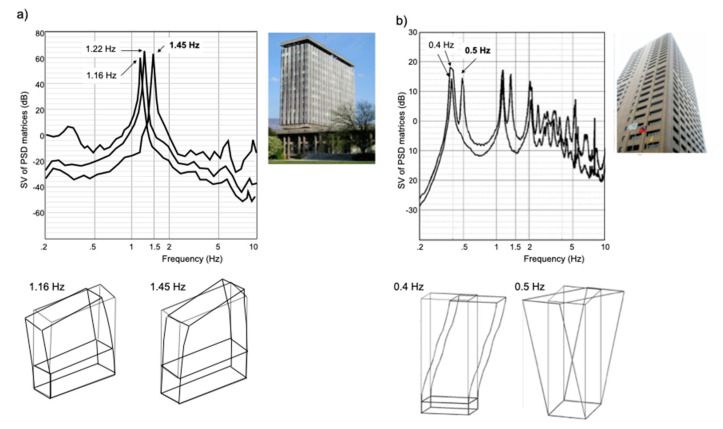
Examples of operational modal building responses using ambient vibrations by Frequency Domain Decomposition. (**a**) City-Hall building in Grenoble (France). (**b**) Taipo Tower in Taipei (Taiwan). Singular value decomposition and examples of experimental mode shapes including torsion are given with resonance frequencies in translation and in torsion (in bold).

In fact, most studies of ambient vibration-based structural modal analysis observe a torsional mode, which is attributed to identifiable static inherent eccentricity or even without apparent eccentricity (e.g., among many others, [101,102,103]). Under ambient vibrations, operational modal analysis (OMA) allows the extraction of resonance frequencies and their corresponding modal shape, especially when the modes are close and without requiring input information on the design of the structure. The two examples given in Figure 2 correspond to two a priori symmetric structures (X and Y horizontal modal frequencies are very close), but with torsional modes clearly identified by OMA. Michel [104] observed that the centre of torsion of the City Hall building in Grenoble shifted with respect to the centre of mass under ambient vibrations but also that this position fluctuated around the average centre over time. This fluctuation may be the result of a dynamic effect dependent upon the loading amplitude (as also suggested by Ivanović et al. [57]), natural wandering of the rotation component with weather conditions (e.g., [58]), or temporal variations in the rotational ground motion. Further experimental analyses are required to confirm these assumptions.

### 4.2. Numerical Observation

Both numerically and experimentally, the modal characteristics in translation (resonance period Ty with y meaning the translation in reference to previous published works, see references above) and the torsion-to-translation frequency ratio Ω are key criteria for the prevalence of ground motion torsional effects in the response of structures (Table 1). However, the publications on these subjects over the last three decades confirm the need for this prevalence to be refined, as the conclusions are often design-specific or even contradictory. For example, De La Llera and Chopra [60] used experimental data to show that during Californian earthquakes, the torsional effect of ground motion was generally less than 8% for uncoupled horizontal vibration periods Ty > 0.5 s regardless of Ω, and it even reached 40% for short periods (i.e., low-rise buildings) and torsionally flexible buildings (Ty < 0.5 s; Ω < 2/3). This was confirmed by Ghayamghamian et al. [105] for Ty < 0.3 s and Ω < 1, and it is also consistent with the results proposed a long time ago by Tso and Demsey [106] and Tscicnias and Hutchinson [52]. Shakib and Tohidi [107] also compared the influence of Ty and Ω in symmetric and asymmetric buildings. They showed that torsional ground motion is more prevalent in the structural response of symmetric buildings than in asymmetric buildings, especially for Ty < 1 s and Ω < 1, but also for Ω < 1.5 in asymmetric buildings, concluding on the underestimate of the accidental eccentricity (*e_a_*, Equation (2)) provided in codes (as suggested also by [105]). They also showed the strong influence of site conditions for symmetric and asymmetric designs, which are related to soil–structure interactions. When Ω increases (>1), the effect of torsion decreases.

Ghayamghamian et al. [105] also found that the increase in displacement due to torsional ground motion depends on Ω in conjunction with static eccentricity for short-period (low-rise) buildings with large or small plan dimensions. For tall, short-period structures (such as nuclear reactors), Falamarz-Sheikhabadi and Ghafory-Ashtiany [11] also claimed that the effects of torsion on structural loading increase when Ω increases and vice versa; this effect decreases when Ty decreases and static eccentricity increases (i.e., asymmetric building). In asymmetric structures, when Ty increases and Ω decreases, the influence of static eccentricity increases, and the effect of torsional ground motion can be ignored for tall asymmetric buildings. Castellani et al. [87] concluded that the contribution of the rotational component on the response of the structure is more significant for a high-rise building (based on a numerical comparison between a low-rise building Ty=0.48s and a flexible building Ty = 1.56 s), which is a difference that can exceed 20%. They also showed that the higher modes could be neglected and that only the low-frequency portion of the response spectra induces significant rotational excitation.

These observations led the authors to consider β values as conservative or non-conservative. For example, Newmark and Rosenbluth [108], Luco and Sotivopoulos [109], and De La Llera and Chopra [60] showed that considering the effect of rotational ground motion for structures Ty > 0.2 s, β = 0.05 is significant in symmetric structures but not for Ty < 0.2 s and long plan dimensions. Ghayamghamian et al. [105] showed that for Ty < 0.3 s and Ω < 2/3, β = 0.05 underestimates the accidental torsion. According to Shakib and Tohidi [107], β is less than 0.05 regardless of Ty when Ω > 1. On the other hand, β = 0.05 is not sufficient for the design of structures when Ω < 1. Finally, according to Falamarz-Sheikhabadi and Ghafory-Ashtiany [10], β = 0.05 seems to be conservative with respect to the influence of rotational ground motion for asymmetric buildings; i.e., the inherent eccentricity due to the CM/CR shift is prevalent in the structural response. Recently, Rahat Dahmardeh et al. [110] concluded that torsional component can increase the structural displacement and drift ratio up to 36% and 41%, respectively, and they confirmed that β = 0.05 is not sufficient for accidental eccentricity and may lead to unreliable structural responses. In all cases, the increase in displacement due to accidental torsion is greater than that recommended in the design codes. The rapid attenuation of rotational ground motion with distance means that the ratio between rotational and translational spectra is one order of magnitude higher in the near field than in the far field [111]. This near-field condition, where the rotation is strongest, is rather limiting in terms of experimental confirmation because of the lack of instrumented buildings.

### 4.3. Array-Derived Rotation

Pure rotation sensors have only recently become available in seismology and, without reliable direct measurements of strong ground rotations, indirect methods based on translation sensors for engineering purposes should be considered. Several approaches are possible, for example using a single three-component sensor for ground motion, based on body and surface wave propagation (e.g., among others, [18,112,113,114] and based on the hypotheses of plane waves, a horizontally homogeneous medium and the dispersive properties of surface waves. An alternative to the single-sensor method is the use of sensor arrays, with the most popular probably being the computation of spatial derivatives of the horizontal components of ground motion according to a finite difference scheme (e.g., [20,71,115,116,117]. With this method, rotation can be estimated with closely spaced stations for seismic waves with long wavelengths compared to the aperture of the array. Theoretical explanations can be found in Cochard et al. [118], recalling the framework of elasticity of small deformations to define the relations between translational and rotational motions. For torsional rate, this method consists in calculating the differences according to the following equation:

(3) $${\dot{\theta }}_{z}=0.5\left(\frac{\partial {\dot{u}}_{y}\left(t\right)}{\partial x}-\frac{\partial {\dot{u}}_{x}\left(t\right)}{\partial y}\right)$$

where ${\dot{u}}_{x}\left(t\right)$ and ${\dot{u}}_{y}\left(t\right)$ are the relative translational velocities along the x and y directions between two sites, divided by the inter-station distance along the x and y axes. Applied to structures, this rotation is expressed by calculating the difference in translational motion between two sensors located at opposite points on the same floor. When they are aligned in one of the main directions of the building, a simplified equation is as follows:

(4) $${\dot{\theta }}_{z}=\frac{\partial {\dot{u}}_{y}\left(t\right)}{\partial x}=\frac{\partial {\dot{u}}_{x}\left(t\right)}{\partial y}.$$

Equation (4) suggests that the shear strain in plane is infinitesimal (i.e., stiff roof) compared to rotation, and strain gradients are consistent along the two orthogonal horizontal axes of the building. In this case, whatever the considered pair of stations, the torsional rate value is the same. Many examples in buildings apply this experimental method to estimate building torsional rotation (e.g., [74,101,102,119,120,121]). Recently, Lin et al. [122] investigated the comparison between array-derived rotation and point rotation in the 101 Taipei Tower (Taiwan), which is probably the only comparable study, since so few rotation sensors are actually deployed in structures. The results are consistent but raise the question of the inter-station distance used as a function of wavelength. For rotational ground motion applications, the results are only reliable within a relatively limited range of frequencies, depending on the size of the array, which should be at most 1/4 of the wavelength (e.g., [123,124]). In buildings, this condition is limited by the lateral dimension of the structure, its frequency, design, and excitation wavelength (e.g., [125,126]), but it has rarely been extensively tested, and efforts should be made to do so. Other methods exist, which are expressed in terms of a displacement–gradient matrix (e.g., [19,127]) and, more recently, Basu et al. [128] proposed a method involving a large number of sensors. Structure networks are rarely large enough to apply enhanced multi-sensor methods.

Recent developments in seismology, mostly thanks to the development of new rotational instruments, have seen the emergence of sensors such as radiometers, ring-laser sensors, and optical sensors (e.g., [22,26,27,29]), but these are still very marginally used in buildings. However, in the same way as in seismology, instrumental development in rotation may allow the use of new observations, coupled with new signal processing processes that would provide new insights on the rotational response of structures. Some observations, described in the next section, may then open perspectives to refine the principles and foundations of the rotational dynamics of structures, addressing the fields of earthquake engineering and S^2^HM. 

## 5. Rotational Structural Response for a Wide Range of Earthquakes

**Figure 3:**
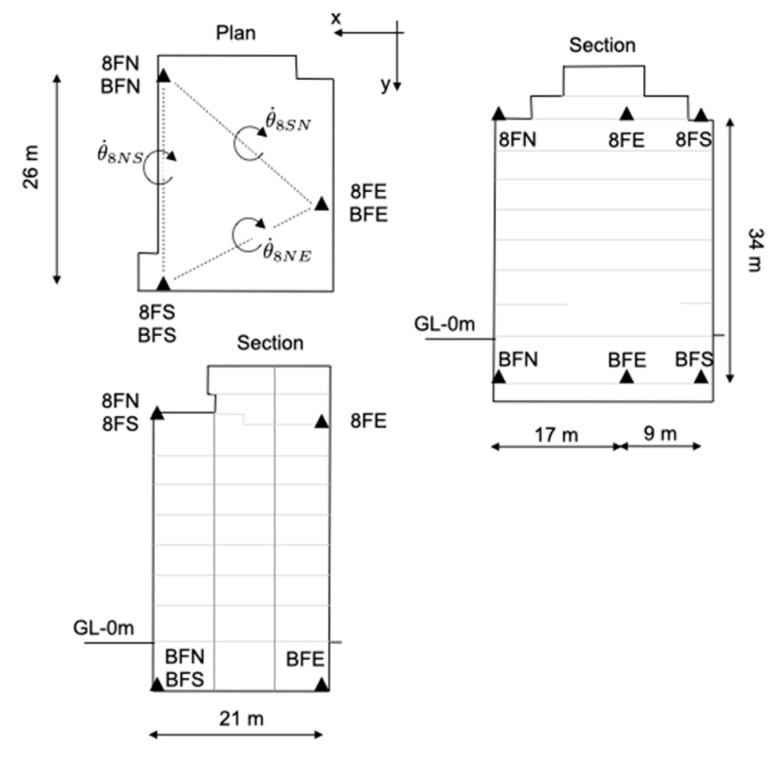
Schematic view of the ANX building and the positions of the sensors deployed at the top and bottom used in this study.

In this section, the data recorded in the ANX building (Japan) are analysed to illustrate the contribution of experimental data to improving our knowledge of the torsional response of structures to earthquakes. Located in Tsukuba, about 60 km northwest of Tokyo (Japan), ANX is an eight-storey steel-reinforced concrete structure [129,130]. Figure 3 shows the plan and cross-sectional views of the ANX building, as well as the location of the sensors whose data were used in this study. The building rests on soft soil overlaid with clay and sandy-clay materials to a depth of at least 40 m (i.e., Vs ≈ 190 m/s—[10]). Its construction was completed in March 1998, and the building has been monitored since 1998 by a network of instruments operated by the Building Research Institute in Japan. The accelerometric sensors used in this study (three at the top and three at the bottom) are oriented along the main horizontal directions of the building. The standard used to reference the components is Floor Number/Plan Position (e.g., 8FN: floor 8, north corner).

### 5.1. Data

This study considers 1630 earthquakes occurring between 1998 and 2018, including the main shock of the great Tohoku Mw 9.0 earthquake of 2011 and its sequence of aftershocks. The data and calculated parameters constitute the NDE1.0 database described in Astorga et al. [131]. The accelerometric data are sampled at 100 Hz. The data correspond to earthquakes of magnitudes between 2.4 and 9.1 and epicentral distances from 1 to 500 km (source: Japan Meteorological Agency). The maximum ground acceleration (PGA) recorded by a nearby free-field station ranges from 0.4 to 279.3 cm/s2, corresponding to peak top accelerations (PTA) of 0.39 to 505.18 cm/s2. The highest PTA and PGA values clearly correspond to the largest earthquake (Mw 9.0), Tohoku, in March 2011, which was located 330 km northeast of the ANX building. All these data were used to compute total structural drifts (calculated as the relative displacement between top and bottom, divided by the height of the building, 34m) ranging from 10^−7^ to 10^−3^.

In a first step, trends and mean values were removed from accelerometric data, and a Butterworth filter of order 2 was applied between 0.1 Hz and the Nyquist frequency. Then, the velocities were calculated by simple numerical integration of the acceleration records. Peak ground velocity (PGV) and peak top velocity (PTV) ranged from 4.4×10^-3^ to 42.4cm/s and from 7.3 × 10^−3^ to 159.6cm/s, respectively.

### 5.2. Rotation Rate 

The rotation rates were calculated according to Equations (3) and (4), considering the pairs of stations at the top 8FN-8FE (θ_8NE_), 8FE-8FS (θ_8ES_), and 8FS-8FN (θ_8SN_), and at the bottom BFN-BFE (θ_BNE_), BFE-BFS (θ_BES_), and BFS-BFN (θ_BSN_), respectively. For example, at the top of the building, rotation rates were calculated as follows:

(5) $${\dot{\theta }}_{8NE}=0.5\left(\frac{{\dot{u}}_{8FEy}\left(t\right)-{\dot{u}}_{8FNy}\left(t\right)}{∆xEN}-\frac{{\dot{u}}_{8FEx}\left(t\right)-{\dot{u}}_{8FNx}\left(t\right)}{∆yEN}\right)$$

(6) $${\dot{\theta }}_{8ES}=0.5\left(\frac{{\dot{u}}_{8FSy}\left(t\right)-{\dot{u}}_{8FEy}\left(t\right)}{∆xES}-\frac{{\dot{u}}_{8FSx}\left(t\right)-{\dot{u}}_{8FEx}\left(t\right)}{∆yES}\right)\;$$

(7) $${\dot{\theta }}_{8SN}=\frac{{\dot{u}}_{8FNx}\left(t\right)-{\dot{u}}_{8FSx}\left(t\right)}{∆yNS}.$$

**Figure 4:**
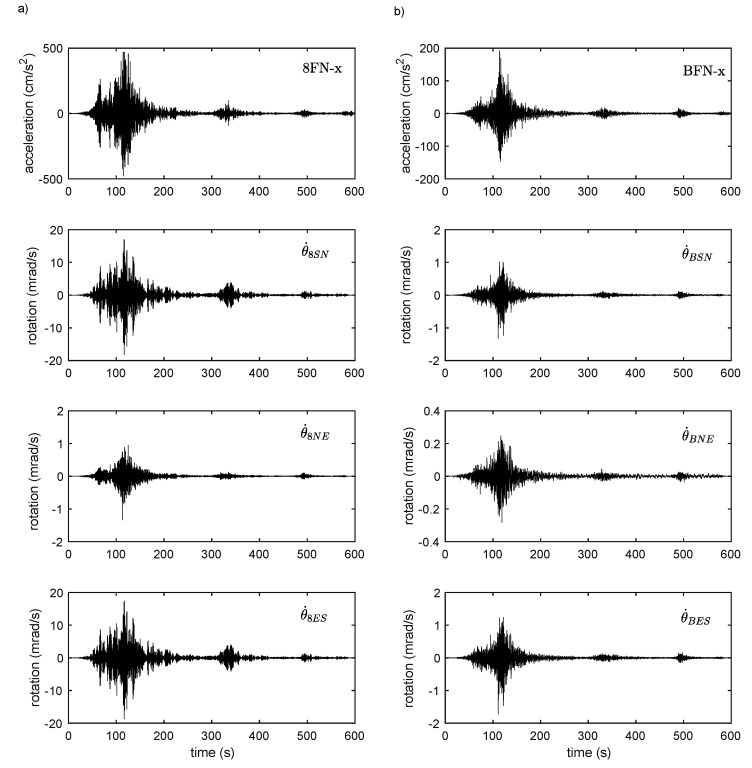
—Example of translational motion recorded during the 2011 Mw 9.0 Tohoku (Japan) earthquake and the array-derived rotational motion (**a**) at the top and (**b**) at the bottom of the ANX building.

**Figure 5:**
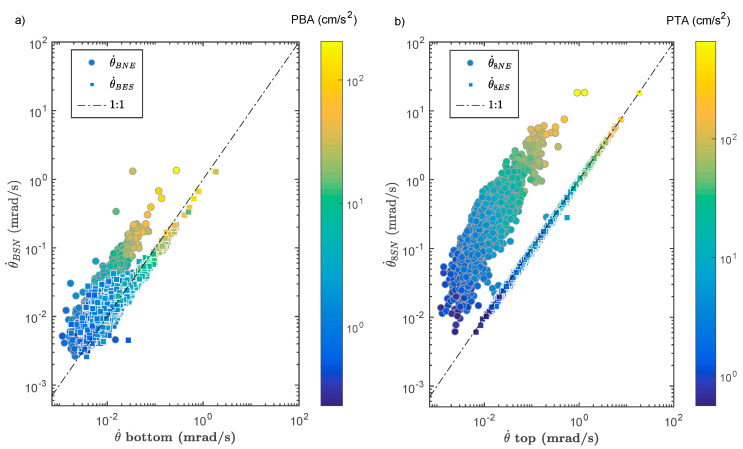
Comparison of the array-derived rotational rate for the ANX earthquake dataset. (**a**) at the bottom (the colour scale corresponds to the peak bottom acceleration (PBA) in cm/s^2^). (**b**) at the top (the colour scale corresponds to the peak top acceleration (PTA) in cm/s^2^). X-label corresponds to the bottom or top rotation rate computed with Equations (6) and (7).

Figure 4 shows the records for the Tohoku earthquake (Mw 9.0, 2011) at the top (4a) and bottom (4b) of the building. The dynamic effect between the bottom and the top is clear, with a factor of ≈2.5 for translational motions and a factor of ≈10 for rotation rates, indicating the prevalence of rotation in the structural response at this level of acceleration. The rotation rates are not identical depending on the formulae used: those with Equations (6) and (7) give equivalent waveforms and amplitudes, whereas Equation (5) underestimates the rotation rate, both at the top and at the bottom of the structure. Under low shear strain, i.e., the structure behaves similar to a rigid body in plane, the three rotation rates should be identical [122], which is not the case here. Figure 5 confirms this observation, for all data, regardless of the loading level. The values of the rotation rates ${\dot{\theta }}_{8ES}$ and ${\dot{\theta }}_{8SN}$ are identical, meaning that the centre of rotation at the top of the structure is at the same distance from the midpoints of the lines between each considered pair of stations. However, the different rotations reflect the fact that the structure does not behave similar to a rigid body in plane, but experiences shear deformation, in relation with the azimuth of the earthquake or the eccentricity of the building. This questions the validity of array-derived methods to estimate rotation, whatever the considered pair of stations and the position of the centre of torsion. In the rest of this study, only the rotational rates ${\dot{\theta }}_{BES}$ and ${\dot{\theta }}_{8ES}$ will be used.

**Figure 6:**
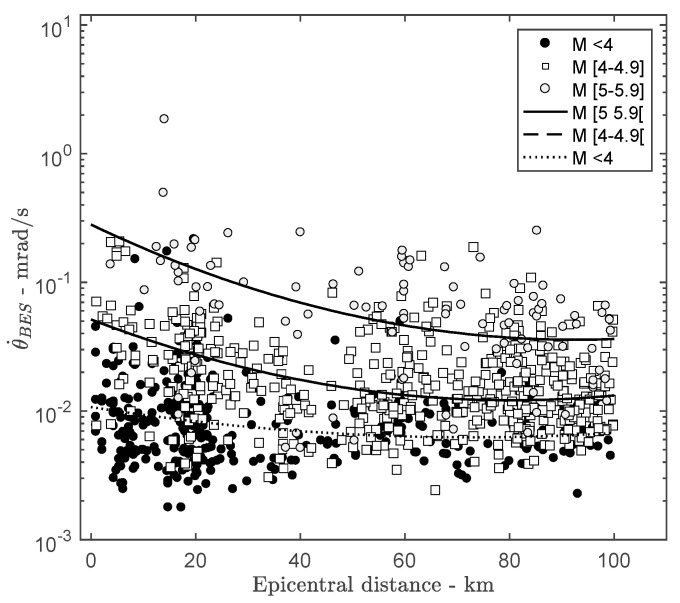
Attenuation of the rotation rate at the bottom of the ANX building with respect to the epicentral distance for several magnitude bins.

Figure 6 shows the rotation ground motion at the bottom of the structure with respect to the epicentral distance for different bins of magnitude. Rotation rate attenuation with distance is observed, as already reported by Bouchon and Aki [28] and Castellani and Boffi [18]. However, the linear fits applied to the data bins show the effect of the source characteristics (magnitude) on the rotational rate attenuation with distance. Data are scattered around the fit, which suggests secondary effects not discussed herein.

### 5.3. Comparison of Rotations and Translations

**Figure 7:**
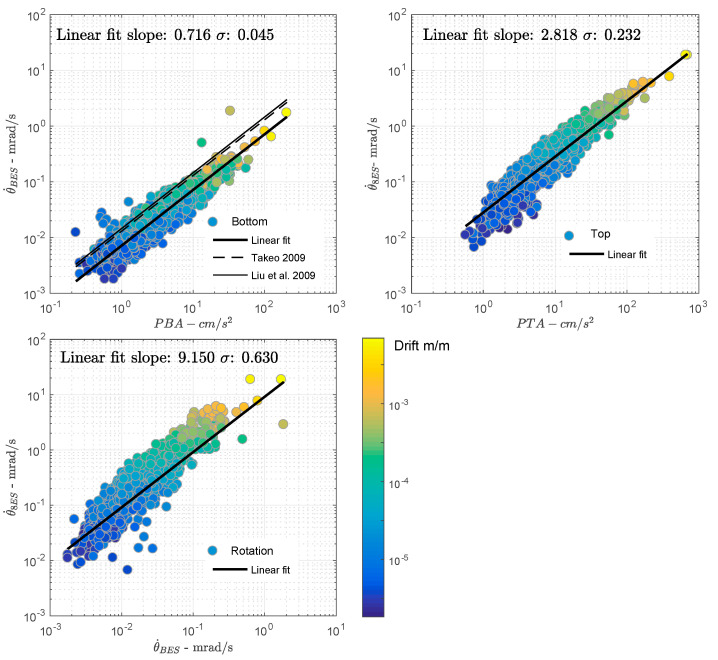
Comparison of the translational (cm/s^2^) and rotational rates (mrad/s) observed in the ANX building: (**a**) at the bottom, (**b**) at the top, (**c**) comparison of bottom and top rotational rates. Thick lines correspond to a linear function (y = ax + b) fitted to the data. σ is the standard deviation of the residual values (observed–predicted).

Figure 7 shows the comparison of translations in cm/s^2^ and rotations in mrad/s. Without further information on the relations between rotations and translations, a function of the form y=ax+b is fitted to the data. At the bottom of the building (7a), the ratio between rotational rate and acceleration is 0.716 with a σ (standard deviation of the residues) value of 0.045, confirming a simple linear relationship. Compared with Takeo [132] and Liu et al. [25], who reported a ratio around 1 in free field, this ratio is lower. In our case, the rotation rate is calculated at the base of the foundations, with definite soil–structure interaction effects. Analysis of the inertial interaction of a structure with shallow embedded foundations is traditionally performed by imposing that the input motion of the foundations is simply that of the free field, thus neglecting the kinematic interaction generated by the travel of seismic waves. This leads to excessive conservatism, because it has been proved that free field motion can be completely filtered by the foundations. With rigid foundations, the kinematic effect is observed if the dimensions of the foundation are equal to or greater than the apparent wavelength in the frequency range of interest, showing reduced motion at the foundation with respect to the corresponding free field [133]. As a result of the high-frequency content of the rotation compared to the translation motion, the kinematic effect may also have bigger effects on rotational motion that could be explored in further analysis thanks to the design of the ANX array. 

**Figure 8:**
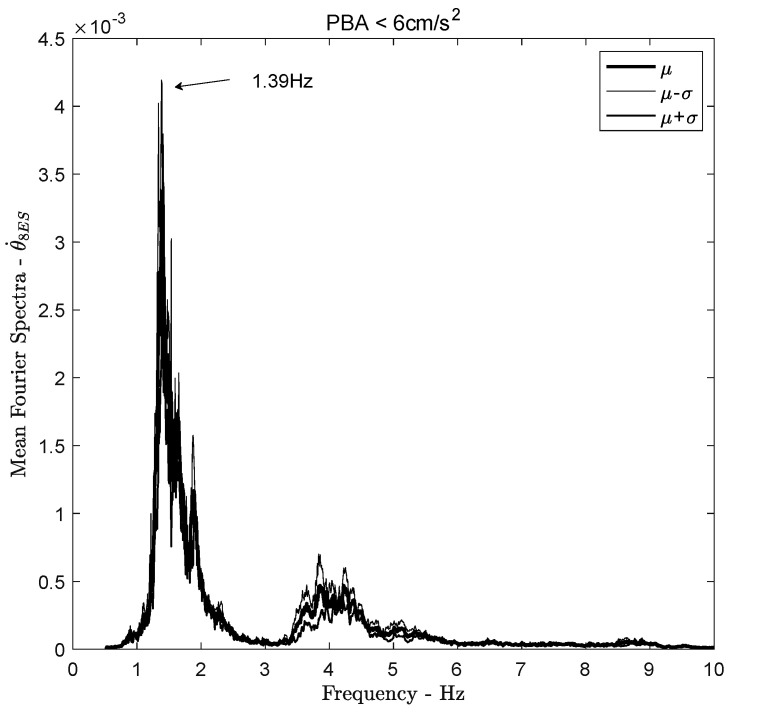
Fourier spectra of the rotational rate computed at the top of the structure. Bold line corresponds to the mean Fourier spectra of earthquakes with PBA < 6 cm/s^2^. Thin lines correspond to the +/− standard deviation Fourier spectra.

Astorga et al. [13,14] showed that the resonant frequency of the translational structure varies over time, from about 1.6Hz in 1998 to 1.0Hz in 2018, as a consequence of structural degradation. Over the most stable period (T2, according to [13]), the translational frequency of ANX is 1.38Hz. Over the same period, the torsional frequency is 1.39Hz (Figure 8), i.e., a wavelength of about 137 m, which is much larger than the dimensions of the foundations, that does not confirm the kinematic effect.

At the top (7b), the relationship is 2.818 with a misfit of 0.232, indicating obvious scattering of the data, in relation to mechanisms leading to the prevalent effect of rotation in the response of the structure. For example, as shown by Astorga et al. [13,14], ANX deteriorates over time with a reduction of the resonance frequency in translation. It would be useful to have further studies to evaluate the effect of this degradation over time on the torsional resonance frequency, as an indicator of damage. Furthermore, the frequency ratio Ω is 1, classifying the building as a torsionally stiff building (Ω > 2/3 and Fy < 2 Hz) according to De La Llera and Chopra [60], i.e., torsion only makes a small contribution to the building’s response. However, Figure 7b shows that rotation is not negligible in the ANX case study; this could be investigated more fully in future studies. 

Finally, Figure 7c compares the rotation at the bottom and at the top of the structure. The dynamic effect of the structure’s rotational response is clearly visible, with a ratio of 9.15. Again, fitting the data by a linear relationship gives a σ value of 0.63, clearly indicating a non-linear amplification of the rotation through the resonance effect between the bottom and top.

## 6. Conclusions and Perspectives

It is now accepted that the rotational behaviour of structures (particularly around the vertical axis) contributes significantly to their seismic response, possibly resulting in significant damage. There are several origins of rotation, including static eccentricity, dynamic or accidental effects, and the contribution of each component remains to be explored. While the importance of rotation has been established, it is a pity to note the rather limited contribution of instrumentation. An increase in the deployment of instruments in (and around) structures including accelerometric sensors, but also rotation sensors, which have seen rapid development over the past decade, is likely to open new perspectives for the understanding and further investigation of this aspect of structural dynamics. With these instrumental perspectives, new applications are expected, in particular for the S2HM with translation and rotation sensors, in connection with the structure capacity by improving the evaluation and characterisation of their actual state (e.g., [37,134]).

This paper is not intended to provide an exhaustive review of all previous studies on the rotational response of structures but rather to focus on a few key components to which the contribution of instrumental data could be beneficial. In particular, the ANX example raises questions on the non-linear rotational response, on how to estimate rotations from array-derived methods, and on the relationship between translation and rotation. Recent methods based on seismic interferometry by the deconvolution technique (e.g., among others [12,13,99]) applied to rotation motion recorded at the bottom and at the top of the structures are envisaged to, for example, separate the CM or CR eccentricity effects from the rotational ground motion effect in the whole torsion response of the building, or to focus on the high-frequency rotation kinematic interaction at the foundation level, for further studies. This example does not allow generalisation to all types of structures defined according to their design or characteristics. However, it does provide a significant set of data, covering a wide range of magnitudes and structural drifts, which should allow further investigation into the contribution of each rotational component to the overall response of the structure.

## Figures and Tables

**Table 1:** Qualitative description of the torsion effects. LR, MR, HR: low-rise, mid-rise, high-rise building. CM: centre of mass. CR: centre of rigidity. e_s_: static eccentricity. e_d_: dynamic eccentricity. e_a_: accidental eccentricity. L: dimension perpendicular to the direction of the seismic motion. Ty: resonance period in translation. Ω: torsion-to-translation frequency ratio. Bldg: building.

Description		Building Features	Qualitative Effect	Refs.
Static e_s_ −CM/CR Shift		asym	Max for Ty	[40,41]
Dynamic e_d_ − e_d_ =α e_s_ with α = 1.5 in EC8		asym Ω = 1	Amplification of e_s_	[46,53]
Accidental e_a_ − e_a_ =βL with β = 0.05 to 0.10 in EC8	CM/CR uncertainties	asym/sym	smaller than β value	[60,61]
	2D trans. ground motion	asym/sym		[61,62]
	Ground motion rotation			
	Differential trans. motion	asym/sym	strong when site effects	[64,65]
	Rotational component			
	**Bldg features consideration**			
	MR-to-HR bldg	Ty > 0.5s	small	[60]
	HR building	Ty > 1.5 s	strong (up to 20%)	[87]
	sym/asym torsionally stiff bldg	Ω>1	small	[107]
	tall and torsionally stiff bldg	Ω > 1 all Ty	increase	[11]
	tall asym. bldg	Ty < 1	decrease	[11]
	LR-to-MR, short period and torsionally flexible bldg	Ty < 1 s – Ω < 1	more prevalent for sym.	[107]
	LR-to-MR, short period and torsionally flexible bldg	Ty < 0.5 to 1s – Ω < 2/3 to 1	strong	[60,105]
	**β consideration**			
	LR-to-MR sym. bldg	Ty>0.2	β = 0.05 significant	[60,108,109]
	LR-to-MR, short period and torsionally flexible bldg	Ty < 0.3 – Ω < 2/3	β > 0.05 for sym. bldg	[105,110]
	torsionally stiff bldg	Ω > 1 all Ty	β < 0.05	[107]
	torsionally flexible bldg	Ω < 1 all Ty	β > 0.05	[107]
	asym. bldg		β = 0.05 conservative	[11]

## Data Availability

The data from Japan presented in this study are available on request from the Building Research Institute (http://smo.kenken.go.jp/).
